# Image-Assisted Microvessel-on-a-Chip Platform for Studying Cancer Cell Transendothelial Migration Dynamics

**DOI:** 10.1038/s41598-018-30776-0

**Published:** 2018-08-20

**Authors:** Cristina Bertulli, Magda Gerigk, Nicholas Piano, Ye Liu, Duo Zhang, Thomas Müller, Tuomas J. Knowles, Yan Yan Shery Huang

**Affiliations:** 1 Cavendish Laboratory, University of Cambridge, Cambridge, CB3 0HE UK; 2 Department of Engineering, University of Cambridge, Cambridge, CB2 1PZ UK; 3 Department of Chemistry, University of Cambridge, Cambridge, CB2 1EW UK; 4 Present Address: Fluidic Analytics Ltd., Cambridge, CB4 3NP UK

## Abstract

With the push to reduce *in vivo* approaches, the demand for microphysiological models that recapitulate the *in vivo* settings *in vitro* is dramatically increasing. Here, we present an extracellular matrix-integrated microfluidic chip with a rounded microvessel of ~100 µm in diameter. Our system displays favorable characteristics for broad user adaptation: simplified procedure for vessel creation, minimised use of reagents and cells, and the ability to couple live-cell imaging and image analysis to study dynamics of cell-microenvironment interactions in 3D. Using this platform, the dynamic process of single breast cancer cells (LM2-4175) exiting the vessel lumen into the surrounding extracellular matrix was tracked. Here, we show that the presence of endothelial lining significantly reduced the cancer exit events over the 15-hour imaging period: there were either no cancer cells exiting, or the fraction of spontaneous exits was positively correlated with the number of cancer cells in proximity to the endothelial barrier. The capability to map the z-position of individual cancer cells within a 3D vessel lumen enabled us to observe cancer cell transmigration ‘hot spot’ dynamically. We also suggest the variations in the microvessel qualities may lead to the two distinct types of cancer transmigration behaviour. Our findings provide a tractable *in vitro* model applicable to other areas of microvascular research.

## Introduction

The need for *in vitro* systems to model the biology and function of microvasculature has driven the development of more physiologically relevant three-dimensional (3D) *in vivo*-like platforms. These systems have been a key facet in the engineering of tissue surrogates^1,2^, providing mechanistic insights into the role of endothelium and microcirculation in both physiology and disease states. In the last decade, microfluidic technology has facilitated the fabrication of a number of devices with incorporated microvessels^3,4^. However, these tend to address highly specific research questions, which are intrinsically linked with their fabrication pipeline, hence the potential to apply these platforms to wider research questions may be restricted by the methods involved in their production^5,6^. Thus, a tool which is advantageous for scientists across various research fields must combine physiological relevance with simplicity of manufacturing techniques^6^. Many existing models are highly valuable at the bioengineering level. However, the aim now is to reduce the complexity in fabricating and using these devices, as well as to provide more quantitative data analysis methods, ultimately making them more accessible and appealing to researchers across disciplines.

Although a variety of techniques to fabricate 3D endothelial vessels inside microfluidic devices have been described (e.g. summarised by Searson and co-workers^4^), most research groups opt for polidimethylsiloxane (PDMS) molding combined with direct hydrogel injection, which provides an excellent base to study mechanistic processes associated with haematological diseases^7^, or critical steps in cancer metastasis^8^. In particular, exit of cancer cells from the blood vessel into secondary organs^9–11^ have been replicated in microfluidic systems employing varying levels of complexity^12–15^. These studies have begun to show that microfluidic platforms indeed recapitulate the 3D *in vivo* conditions more closely than 2D models^16^. Despite this recent progress, and even though microfluidic systems provide a favorable platform to undertake such well-controlled experiments, statistical analysis of cellular dynamics is rare.

Here we describe the design of a microfluidic device, in which an *in vitro* vessel of rounded cross-sectional geometry and an endothelium-extracellular matrix interface is obtained from simple, reproducible device preparation procedures. The artificial vessel is designed to mimic the physiological microvessel structures where cancer cells perform transmigration^4^, from a vessel lumen to the surrounding extracellular matrix (ECM). Standardized geometry of the microfluidic device provided us with a great opportunity to develop a pipeline that couples the microfluidic-based microvessel with an image analysis platform, which allows tracking of the transendothelial migration processes. Supported by the experimental and analysis capability, we defined three spatial environmental regions to evaluate transendothelial migration dynamics: the microvessel lumen, the endothelium/ECM interface and the 3D gel matrix. Image stacks of each time point were simplified into a 2D projection, which were then used to extract useable information for a 3D environment, not possible with 2D imaging. This method was also resistant to the issues of focal plane drifting during live-cell imaging. Using the designated system, we were capable of quantifying the cellular dynamic events associated with distinct regions within the 3D microenvironment.

## Materials and Methods

### Fabrication of the microvessel-on-a-chip

**Figure 1:**
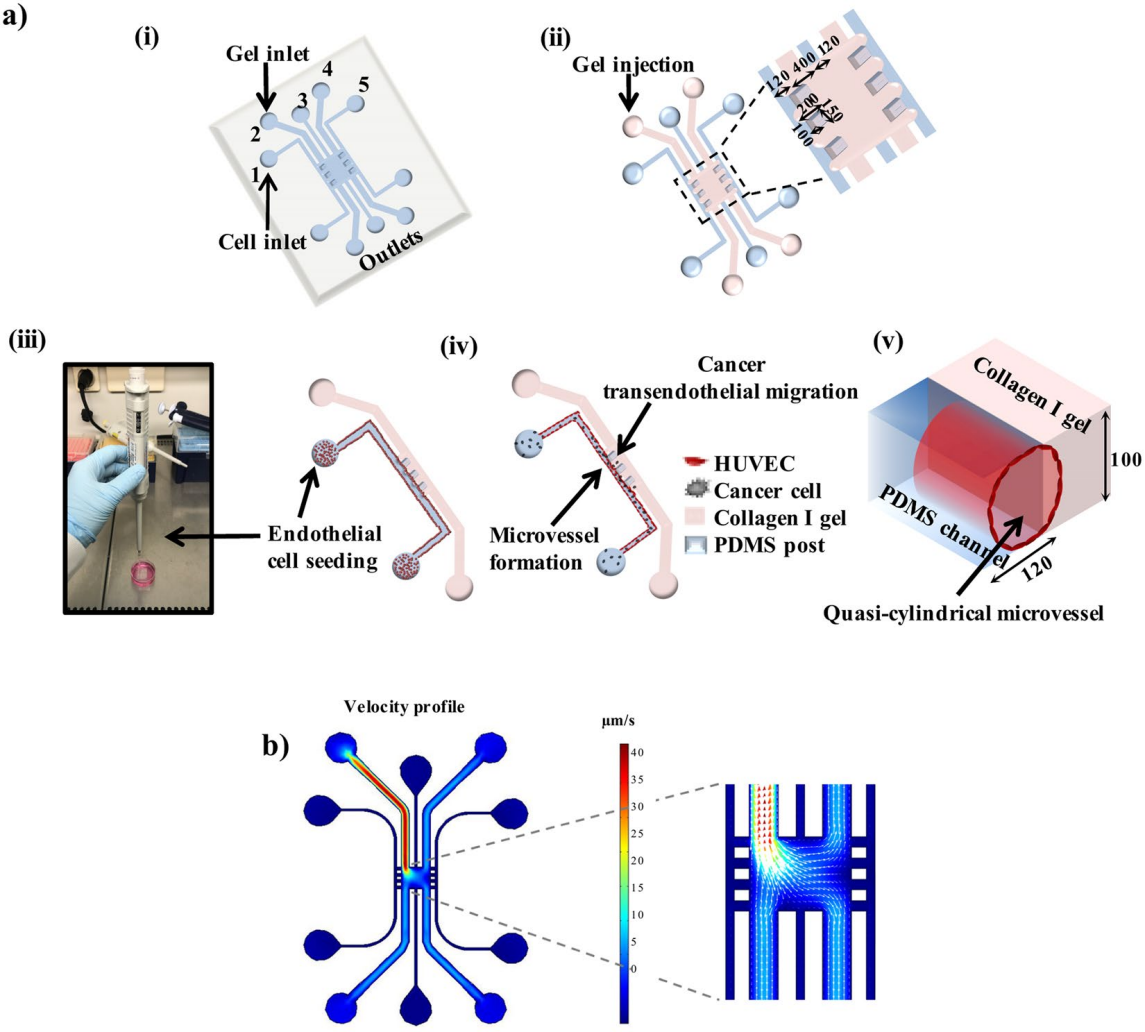
Microfluidic design and microvessel fabrication. (**a**) (i) scheme of the microfluidic device; (ii) gel injection leads to two vacant side channels, and a central 3D gel chamber; all dimensions in µm; (iii) seeding of endothelial cells at a side channel forming a microvessel; (iv) injection of cancer cells into the microvessel for the transendothelial migration studies; (v) a rounded vessel formation due to the confined channel dimension; all dimensions in µm. (**b**) Numeric simulation of the flow velocity profile for collagen gel injected into the microfluidic device. Flow direction and velocity magnitude are represented by the white arrows.

The microfluidic device described in this work is shown in Fig. 1. It consists of two outermost side channels (120 µm wide, 100 µm high), as well as three middle channels (two of which are 400 µm wide and 100 µm high, and one channel that is 120 µm wide and 100 µm high) merging in the central region of the device, which can contain collagen I gel which acts as 3D ECM. Here, one of the two outermost channels was used for endothelial cell culture. The outermost side channels are connected to the central region of the device through the gaps between pillars. The microfluidic master was fabricated using soft lithography. A negative photoresist SU8 (MicroChem) was spin-coated on a 6” silicon wafer and the mask was then patterned by UV exposure. The photoresist was developed to eliminate the non-illuminated parts and the final master is obtained. The channels were fabricated by molding PDMS on the master. PDMS (Sylgard 184), at 10:1 (w/w) ratio of elastomer to curing agent, was mixed thoroughly, poured onto the master and desiccated to remove any air bubbles formed during the mixing process. PDMS was then cured for 5 hr at 65 °C. Afterwards, PDMS was peeled off, and access ports of 0.75 mm in diameter were made. A bottom PDMS layer (1 mm thick) was prepared by curing PDMS, under the same conditions as above, in a glass Petri dish and cutting out a rectangular piece to cover the top PDMS part. Foreign particles were removed from the PDMS surfaces using transparent adhesive tape; the PDMS pieces were soaked in ethanol for 18 hr to dissolve non-cross-linked PDMS residuals. The PDMS surfaces were being dried off at 50 °C for 1 hr and they were bound to a 1 mm thick PDMS layer by air-plasma treatment (Femto Science, 15 s, 25 sccm, 10 power), forming a microfluidic device.

The process of vessel formation is shown in Fig. 1a; all the fluidic injections and removals were performed using 2–20 µL pipette tips. After plasma treatment, poly-d-lysine (PDL, Sigma, P7280-5MG, 1 mg/ml in deionized water (DIW, Millipore)) was injected inside the channels. PDL coating was performed to facilitate the adhesion of the collagen gel, which can be inserted afterwards, to the PDMS surfaces of the device. After PDL injection, DIW was added to the surrounding of the device to avoid evaporation of the solution from the channels, and the device was left inside the cell culture incubator for 5 hr. Then, channels are washed multiple times with DIW to remove any excess, unbound molecules. After the last washing, the solution inside the channels was completely removed and the device was placed at 50 °C for 20–28 hr to restore hydrophobicity of the bonded surfaces. Next, collagen type I gel (Gibco, A1048301) was prepared (as described below in 2.2) and carefully injected from the access port of one of the channels directly facing the side channels. Vertical interfaces were created at the gaps between the pillars separating the side channels from the central region. 10 min after gel insertion, drops of medium were added on top of the access ports of all channels, except for the two outermost side channels. After gel cross-linking, collagen IV (Sigma, C5533, 1 mg/ml in DIW) was inserted into the outermost side channels to create a second layer of coating of the channel surfaces which is suitable for HUVEC (Human Umbilical Vein Endothelial Cells) attachment^17^. The coating was performed at room temperature for 3 hr. After collagen IV coating, the outermost side channels were washed several times with cell culture medium to remove any residual, unbound collagen. Subsequently, endothelial cells were seeded inside one of the outermost side channels and the device is stored inside the incubator for about 1–2 hr to allow the cells to attach to the bottom surface of the channel. A second seeding of endothelial cells was then performed and the device was put upside-down to allow cells to attach to the top surface of the channel. 1 hr after the second seeding, the device was put back in the initial position for a confluent layer to form.

It is worth acknowledging that the devices can be stored after PDL coating, as well as after collagen I and collagen IV injection, at room temperature and inside the incubator, respectively. The devices stored after PDL coating can then be used within one week from their preparation, and they can be kept with collagen I and collagen IV at 37 °C for at least 18 hr before cell seeding.

### Collagen I gel preparation and gel injection simulation

Deionized water, collagen I rat protein, ×10 PBS (Gibco) with phenol red and 0.5N NaOH (Sigma-Aldrich) solutions are kept on ice. Using a large orifice pipette tip, 27.2 µl of collagen was added to a 1 ml tube, which was then also kept on ice. 5 µl of ×10 PBS with phenol red and 3 µl of 0.5N NaOH was added to a new 1 ml tube and mixed. 5.4 µl of DIW was added and mixed. This solution is pipetted out into the tube containing collagen solution and the entire mixture is pipetted up and down and swirled, until the gel appears to be uniform in color. Following the above procedure, the collagen gel had a final concentration of 2 mg/ml and a pH around 7.5. Afterwards, the gel was injected inside the microfluidic device and cross-linking occurred at room temperature for 1 hr.

COMSOL Multiphysics 5.0 (COMSOL Inc., MA, USA) software was used to model the steady state laminar flow of collagen gel inside the microfluidic channels. A 2D simulation was generated by importing the device geometry from the AutoCAD design file into the COMSOL laminar flow module. The density of the collagen gel was given as 2 mg/ml and the dynamic viscosity was set as 6 mPa·s. Velocity at the inlet was defined as 50 µm/s and the pressure at the outlets as 0 µm/s. Flow was assumed incompressible and flow velocity was estimated based on the Navier-Stokes equations. All boundaries were set as no-slip.

### Cell culture

Endothelial cells, HUVECs (PromoCell, C-12253), were cultured in growth cell culture medium (Lonza, CC-3162) and used at passage 6 in all experiments. Green fluorescent protein (GFP)-expressing breast cancer cells, MDA-MB-231 and LM2-4175 (obtained from Dr Joan Massagué, Sloan-Kettering Institute), as well as human endothelial cells, EA.hy926 (ATCC, 2922) were cultured in Dulbecco’s Modified Eagle’s Medium (DMEM, Invitrogen) supplemented with 10% Fetal Bovine Serum (FBS, Sigma-Aldrich) and 1% Penicillin/Streptomycin (Sigma-Aldrich). Cells were grown under 5% CO_2_ at 37 °C. Before seeding endothelial cells inside a microfluidic device, HUVECs were starved for 4 hr by changing growth cell culture medium in the flask to medium of reduced serum content (0.5% FBS). Afterwards, HUVECs were harvested and re-suspended in the cell starving medium (at a density of 3 × 10^7^/ml) and the cell suspension was mixed by pipetting the solution up and down; this process is required to avoid cell clumping. A similar protocol was used for EA.hy926 cells – medium was changed to that of reduced serum content (2% FBS) 4 hr before the cell seeding. 3 µl of the cell suspension was injected inside the cell culture channel by manually decreasing the pipette volume; therefore, the cell seeding was performed at a very low flow rate (~0.2 µl/s), which was necessary to obtain a uniform distribution of cells inside the channel. In order to avoid any back flow of cell solution inside the channels, the device must be submerged in medium. For transendothelial migration experiments, MDA-MB-231 or LM2-4175 cells were suspended in the endothelial starving medium at a density of 4 × 10^6^/ml. The same seeding technique as described above was used for cancer cell seeding.

### Immunostaining

Cells were fixed using 4% para-formaldehyde (PFA, Sigma-Aldrich) and permeabilized with 0.2% Triton 100X (Sigma-Aldrich) in phosphate buffered saline. Non-specific antibody interactions were blocked by incubating the cells with 4% albumin from bovine serum (BSA, Sigma-Aldrich, A7906) in PBS for 1 hr. For nuclear and cytoskeleton staining, Hoechst 33258 (Sigma, 94403) and Phalloidin Alexa Fluor® 488 (Life Technologies, A12379) were used at 1:10000 and 1:400 dilutions in 1% BSA/PBS, respectively. Hoechst and Phalloidin were incubated with the cells for 50 min at room temperature. For the staining of endothelial adherens junctions, the primary antibody, goat anti-VE-cadherin C-19 (Santa Cruz Biotechnology, sc-6458), was used at 1:30 dilution in 1% BSA and 5% donkey serum (Sigma, D9663) in PBS and incubated with the cells for 1 hr at room temperature. Afterwards the secondary antibody, donkey anti-goat Alexa Fluor® 633 (Life Technologies, A21082) at 1:1000 dilution in 1% BSA/PBS, was incubated for 50 min with cells. The devices were then stored in PBS at 4 °C and fluorescence imaging was performed within 2 days from staining. Subsequently, fluorescent images were acquired by Leica SP5 confocal microscope, using a z-stack with a 2.5 μm step size. Three-dimensional reconstruction of the confocal image slices was performed with ImageJ.

### Time-lapse imaging of cancer transendothelial migration

Cancer cells were injected inside the cell culture channel and the trans-endothelial migration through the endothelial barrier into the collagen gel was investigated. Time-lapse experiments and z-stack imaging were performed using an environmentally controlled chamber (set at 37 °C, 5% CO_2_) attached to the confocal microscope. The 488 nm Argon laser was used for the imaging. In each experiment, the time-lapse imaging of one microfluidic device was performed and 1–2 fields of view (samples) of the channel were acquired. For each field of view of the channel (120 μm wide, 413 ± 8 μm long), a z-stack (over the entire channel height of ~100 μm, 1.5 μm z-step) was acquired every 20 min, over 15 hr. For the HUVEC system, a total of 3 time-lapse experiments were performed for the reference system (cancer cells in the channel facing the collagen gel, without endothelial cells), resulting in 5 samples acquired (two times two fields of view (FOV), one time one FOV); 3 time-lapse experiments were performed for the microvessel system (cancer cells in the channel with endothelial cells), resulting in 6 samples acquired (three times two FOV). In each sample, behavior of 10–50 cells was monitored over 15 hr. The area of endothelial barrier facing the collagen matrix (i.e. accessible area for cancer transendothelial migration) is of the size of (150 × 100) μm^2^.

### Image analysis of the transmigration dynamics

**Figure 2:**
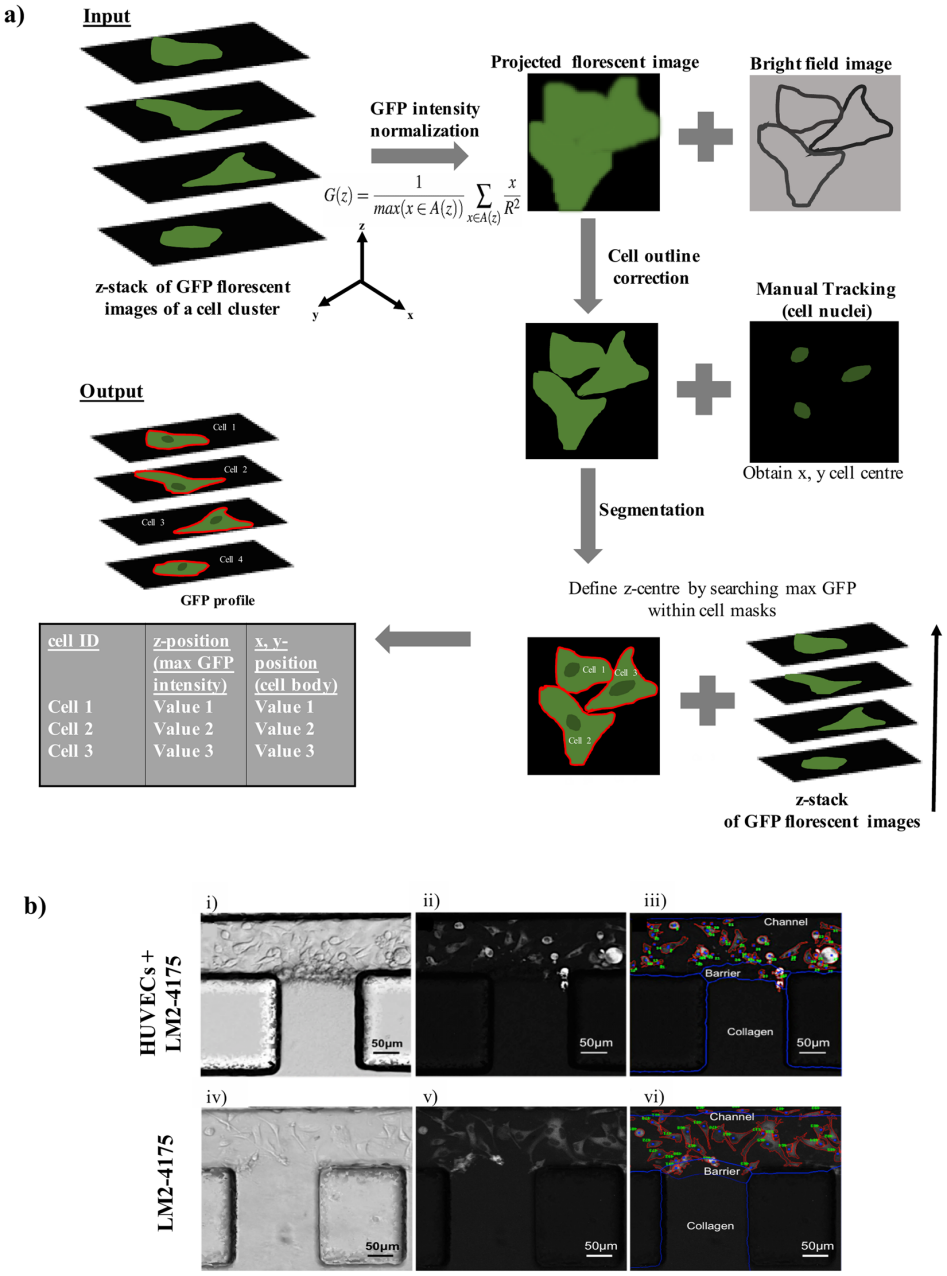
Image analysis for studying transendothelial migration in a microfluidic system. (**a**) Consecutive steps of image processing. (**b**) (i–iv) Bright field images of the microvessel and reference system respectively, at a particular time point and z-level. (ii–v) Fluorescent images of the cancer cells at a particular time point, inside the microvessel and reference system, respectively. (iii–vi) Segmentation of the projected cancer cell areas (red contours) identified by cell number, in the microvessel and reference system, respectively. The blue lines define the edges of the defined regions. The thickness of the barrier (about 40 μm) is due to the curvature of the collagen interface formed at the gaps between the pillars.

An image analysis program was written in house to pre-process the time-lapse images and create a database to automatically classify cell interactions with the regional microenvironments. This novel program utilizes several open-source applications, such as ImageJ and CellProfiler, and merges them with focus stack (z-stack) image analysis to enable cell tracking and segmentation. The data for our experiments use brightfield (or transmission) images and GFP fluorescence images. Figure 2a presents the consecutive steps of image analysis process. Intensity of GFP expression of the z-stack images acquired during the time-lapse experiments was projected onto one x-y plane at each time point; thus the projected cell area, that is the 2D projection of the 3D cell shape, was obtained at each time point. Simultaneously, brightfield images were used for correcting the cell outline, allowing the program to recognise individual cell bodies Afterwards, the ROI manager plugin of ImageJ, applied to the bright field images of the z-stacks, was used to define regions of interest (ROI; channel, barrier, gel) within each field of view (sample). The Manual Tracking plugin of ImageJ, applied to the sequence of images, was used to track the position of cancer cell nuclei over time. Targeting the cell nucleus while tracking cell movements is crucial, to enable the image analysis software (CellProfiler) to perform the segmentation of the cancer cell. During segmentation, the cell shape is separated and differentiated from the background. Segmentation allows us to extract the cell shape features in the previously defined regions at each time point. Furthermore, the z-coordinate of each cancer cell was calculated by finding the z-level of the maximum GFP signal within the projected cell area. Cell segmentation combined with maximum GFP analysis determines the cell centre. All of the obtained information were used to describe cell behavior in the presented system.

### Permeability measurement

Permeability measurement and calculations adapted from Funamoto *et al*.^18^ was used in our work. The microvessel permeability was assessed by observing the diffusion of 70 kDa FITC-dextran (Sigma Aldrich, 46945; 7 mg/ml in PBS, diluted further 1:9 in cell culture medium) from the microvessel channel into the collagen gel. The diffusion experiments were performed in the environmentally controlled chamber (set at 37 °C, 5% CO_2_) of a fluorescent microscope. The dextran solution was perfused inside the microvessel channel and images were acquired every 30 sec over 30 min. Obtained fluorescent images were analyzed using commercial software (ImageJ).

## Results

### Microvessel device design

The microfluidic platform we developed allows formation of vertical collagen interfaces (facing the cell culture channels) by retaining the collagen gel in the central region of the device after injection. The confinement of the gel in this location strongly relies on the channel geometry and on the presence of pillar arrays between the central region of the device and the cell culture channels. The use of pillars between channels has been adopted in previous studies to retain collagen gel in particular locations within microfluidic devices^19–21^. However, in our study a specific device design was required to create a continuous interface along the channel, where wide spacing between the pillars allows cell-matrix interaction. Retention of the gel in the central region of the device, referred to as “gel trap”, was demonstrated by performing a COMSOL simulation of the collagen gel velocity profile during injection inside the microfluidic device (Fig. 1b). These simulations confirmed the presence of a “gel trap”, illustrated by zero flow velocity and stagnant flow at the left and right pillar arrays, and also indicated no leakage of gel into the outermost side channels used for cell culture. Instead, the flow in the central region of the device was not found to be influenced by the presence of the pillar arrays. Thus, vertical collagen interfaces could be easily formed at the gaps between the pillars. The design of the device restricts occurrence of transendothelial migration sites to the designated areas of the vertical barrier between the pillars, enhancing the imaging and analysis capability.

### Image-assisted platform for tracking cell behavior over time

An important part of live-cell image analysis is the thorough measurement and tracking of cell dynamics during an experiment. Therefore, accurate cell segmentation is crucial to extract useable information from imaging, providing representative data of cell movements and shapes. A key limitation in widely used software is the inability to account for consistent features located in 3D data. 3D image data, such as from confocal microscope imaging, contain information about environment on many focal planes. Objects can appear blurred or in focus depending on the plane. Consequently, features that are useful for segmentation - dark edges, uniform bright interiors, and other, such as fluorescent markers expressed within cells - are sensitive to fluctuations in focus or the movement of objects vertically in the environment. This precludes consistent segmentation of a cell. Moreover, although having cells with both cytoplasm and nuclei fluorescently labelled would make the process much simpler, cells commonly used for experiments where live-cell imaging is performed are usually tagged with just one type of fluorescent marker (*e*.*g*. green fluorescence to show cytoplasm). Without well-defined nuclei, two (or more) cells which merge into one cluster would make cell segmentation difficult.

To improve segmentation and the ability to extract quantitative cellular dynamics, our method used information from both the brightfield and fluorescent GFP images obtained from time-lapse confocal microscopy. Due to internal cellular distribution of the GFP, the central regions of a cell (not including the nucleus) are highlighted, so it can be used to locate the general bulk of the cell. The brightfield, on the other hand, shows the edges of the cell more clearly, but only if the cell is in focus (at the correct level). Edges of the cell start to fade along any cell protrusions. In this study, any useful segmentation done must have included accurate outlines of elongated cells to determine cell motility and behavior. Our algorithm used the bulk of the cell visible in the GFP to locate the level in the data needed to ensure consistently clear dark edges in the brightfield, yielding the cell shape in focus. The GFP and brightfield data represented the same physical space. This correspondence was exploited to allow information about the GFP channel to aid searching for features in the brightfield. The correct edges were located by building a vertical GFP intensity distribution for each pixel and selecting pixels from the brightfield data match the level of the distribution peak. Examples of pre-processed images, as well as cell segmentation in different environments, are shown in Fig. 2b. It is worth acknowledging that although the cell lines we used are labelled with only one marker, it is possible to recognize cell nucleus (where the GPF labelling is absent) and therefore perform cell tracking. As presented in ii and v, the cell nuclei are dark (usually circular) spots inside brighter bulk of cells.

The method depends on several parameters, such as R, the radius of the linear smoothing kernel; ΔZ, the level correction of the edges; and Σ, the size of the Gaussian smoothing in 3D. The algorithm is mostly invariant under these parameters, but can be adjusted using ΔZ depending on the type of edges needed for segmentation. Ideally, these parameters would not have been chosen, they would simply be used to explore the data available. In this method, a lot of data are discarded, leaving the level-corrected images. Small shifts in the images, due to movements of the microscope stage during the experiments, are corrected by recognising constant features in the images, such as the pillar corners. The final segmentation presents an error of about 15% on the projected cell area of each cell and an error of about 2 μm (in both x and y direction) in the location of the cell nucleus.

### Formation of the HUVEC microvessel lumen

**Figure 3:**
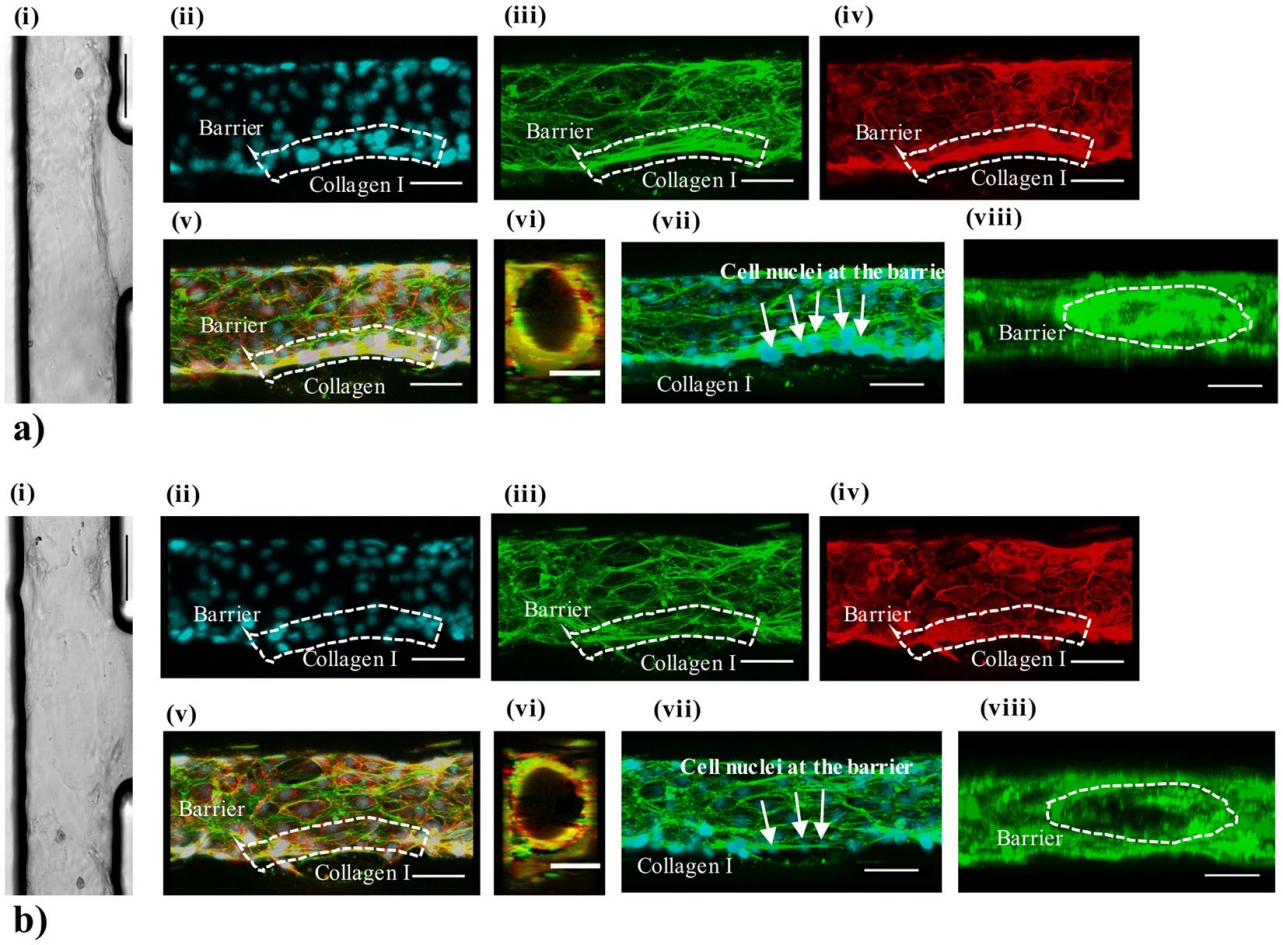
Microvessel lumen formed by HUVECs in contact with collagen I gel. (**a**) Example images of a well-formed 3D microvessel. (i) bright field image; (ii) immunofluorescence image of the nuclei by Hoechst staining; (iii) image of the cytoskeleton by Phalloidin staining; (iv) image of the junctions formed between the cells by VE-cadherin staining; (v) image of three stainings merged; (vi) 3D reconstruction of the microvessel cross-section (using a merged image of Phalloidin and VE-cadherin staining) at a location along the vessel lumen (y-axis); (vii) merged image of the nuclei and cytoskeleton stainings; (viii) 3D reconstruction of the microvessel at a location along the vessel lumen facing the collagen matrix (x-axis). (**b**) Example images of a vessel characterized by weak F-actin expression, presenting the same data sequence (i–viii) as (**a**). All scale bars: 50 µm.

Figure 3(a,b) shows the transmission and immunofluorescence images of two categories of typical microvessels, summarised from over 10 independent preparation runs. For both categories, endothelial layers were observed to form a confluent layer along the bottom and top of a channel, when observed with a transmission microscope prior to cell fixation. Immunofluorescence staining shows that the two microvessels present similar levels of positive staining for F-actin, and VE-cadherin glycoproteins at the cell junctions, along the microvessel channel (see a (ii–v) *versus* b (ii–v)). Three-dimensional (3D) reconstruction of the fluorescence images demonstrates that the lumen of microvessel shows an almost circular cross section (~100 µm in diameter). The cross-sectional view reveals that the endothelial cells round off the corners of the rectangular channel. This phenomenon occurs most likely due to the dimensions of the channel, which are similar to the cell size, and is consistent with previous studies with HUVECs in PDMS micro-channels of rectangular cross-section (50 × 50 μm or 30 × 30 μm)^7,22^. Although the two categories of microvessels appear to be similar under top-down imaging acquisition, a noticeable difference is seen for a reconstructed z-plane of the microvessel. In particular, a higher level of F-actin staining is present at the endothelial wall interfacing the collagen gel, when comparing the first category (i.e. a (viii)) to the second category (i.e. b (viii)). Overall, surveying these 3D reconstructed fluorescence images of the HUVEC microvessels produced at different experimental runs, we suggest that a simple top-down view of the vessel may fail to reflect the variation in vessel qualities.

To test the barrier function of the formed microvessels, we measured the vessels’ permeability to 70 kDa FITC-dextran. An analytical model adapted from Funamoto *et al*.^18^ was used to extrapolate the permeability values of the vessel barrier interfacing the collagen gel. As shown in Fig. SI.1(a,b), the time-lapse fluorescence intensity measurement yields a value for the HUVEC vessel permeability to 70 kDa dextran to be 12 ± 2.4 × 10^−6^ cm/s (n = 3). The analysis was performed for the first 15 min duration of imaging since beyond 15 min, the geometry of the device (e.g. pillars and the limited size of the gel chamber) breaks down the ‘perfect sink’ condition assumed in the calculation. Comparing the HUVEC microvessel permeability to other literature reported systems in Fig. SI.2c, we suggest that the vessel integrity formed in our system is acceptable.

### Cancer transmigration behaviour and endothelial barrier function

**Figure 4:**
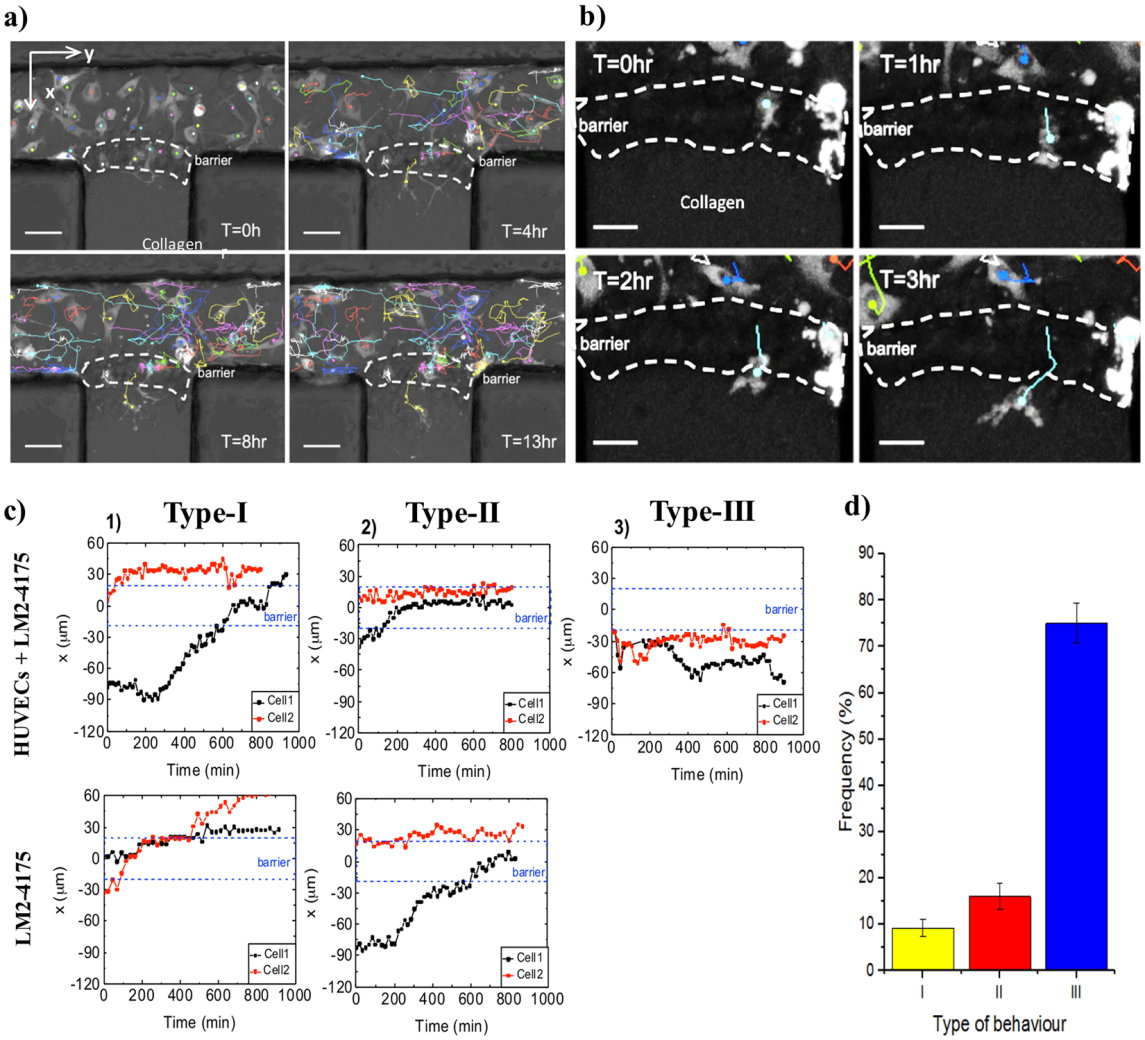
Distinct patterns of cell dynamics observed in the defined environmental regions during the transmigration process. The coordinate axes were chosen as follows: x-axis perpendicular to the barrier towards the collagen; y-axis along the channel; z-axis from the bottom to the top of the channel (*i*.*e*. against the action of gravity). (**a**) Selected frames from time-lapse imaging after bright field subtraction. The trajectories of LM2-4175 cells are shown. The position of the endothelial barrier (about 40 μm thick) is marked. Scale bar: 50 μm. (**b**) Selected frames from time-lapse imaging after bright filed subtraction, showing a cancer cell (light blue trajectory) transmigrating the endothelial barrier, from the vessel lumen into the gel matrix. Scale bar: 25 μm. (**c**) (Top row) x-position over time of two particular cancer cells inside the microvascular system; (bottom row) x-position over time of two particular cancer cells inside the reference system. (**d**) Frequency of occurrence of each one of the three cell behaviors observed in the presented microvascular system. A total of four samples were analyzed (cancer cell count: 13, 15, 20, 26). Error bars: SD.

**Figure 5:**
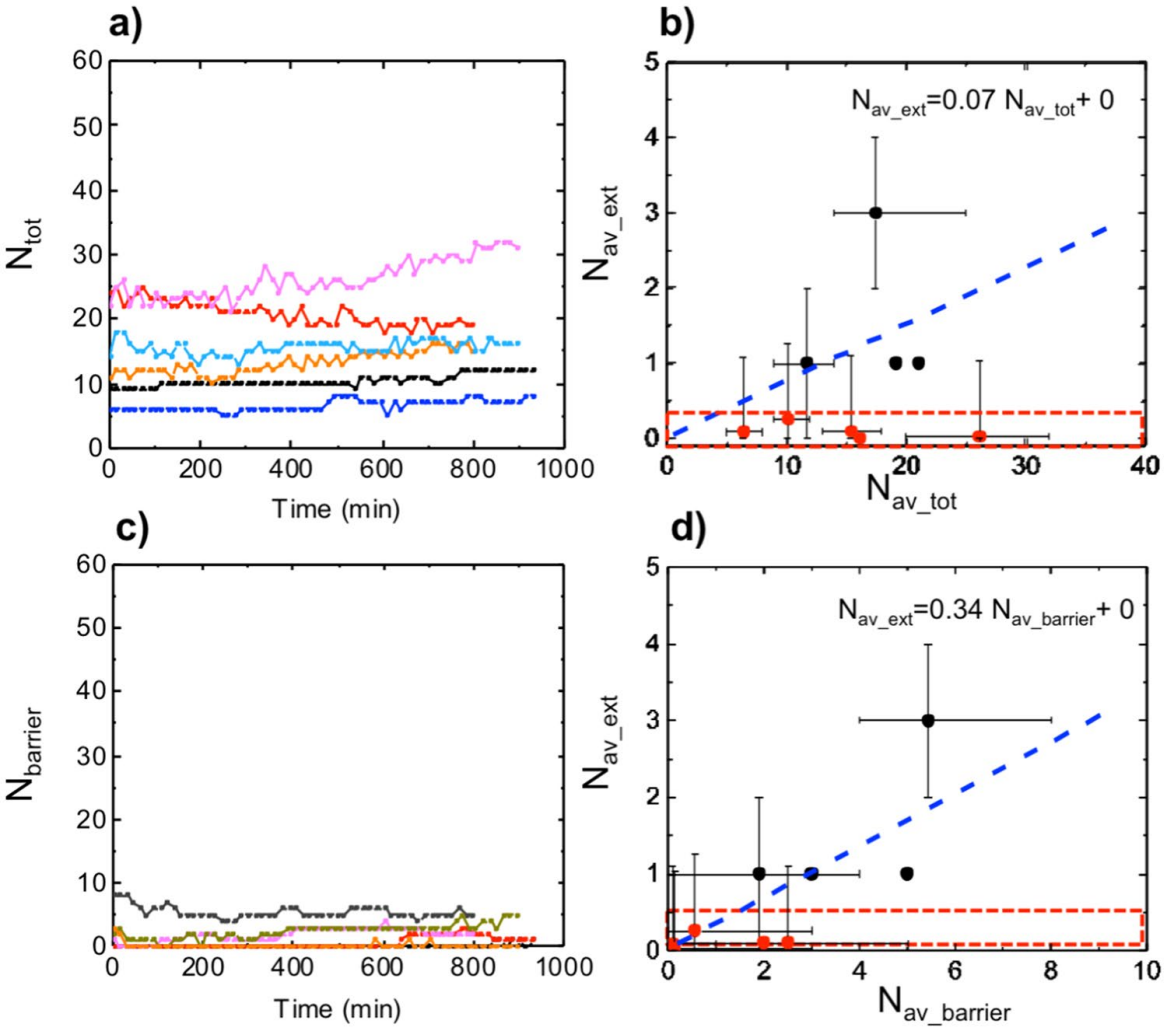
Cell number-dependent transendothelial migration in a microfluidic system. (**a**) Total number of cancer cells present in the field of view over time, for six samples. (**b**) The average number of cells which transmigrated through the endothelial barrier *vs* the average number of cancer cells in the channel and at the barrier, for each sample. (**c**) Number of cancer cells present at the barrier over time, for five samples where cells migrated to the barrier. (**d**) The average number of cells that crossed the endothelial barrier *vs* the average number of cancer cells at the endothelial barrier, for eight samples. The error bars indicate the maximum and minimum values of the fluctuation in the cell number over time. The red box indicates that transendothelial migration did not occur. The data points indicating transmigration events (black dots) were fitted with a straight line with intercept set to zero; the equation of the fitting line is indicated.

To test the described in 3.3 theory, and model transendothelial migration events in our microfluidic platform, LM2-4175 breast cancer cells were injected into the microvessel lumen. Extravasation of cancer cells from the circulation to a secondary site in a distant tissue is known to be a critical step for metastasis^23^. Extravasation involves a cascade of events consisting of cancer cell arrest on the endothelium and cancer cell transendothelial migration and invasion into the surrounding matrix^24^. LM2-4175 is known to efficiently extravasate in metastatic models *in vivo*^25^; therefore, this cell line was chosen as a model for our *in vitro* experiment. The cancer cell behavior was monitored by time-lapse microscopy over about 15 hr, and analyzed using the tracking algorithms introduced earlier. Cellular motility was investigated in different regions which are broadly separated and classified as: the bulk liquid-phase inside the microvessel lumen (channel); the vertical layer of endothelial cells forming the vessel wall or facing the gel matrix in the endothelial-free model (barrier); the bulk of the gel matrix (collagen). Preliminary visual inspection revealed distinct patterns of cell dynamics present when cancer cells moved across these regions during TEM. Figure 4a shows examples of selected frames from the time-lapse imaging, where cancer cells are visible and their trajectories, projected on the x-y plane, are shown over time. In this case, while majority of the cancer cells remained inside the vessel lumen, one cancer cell transmigrated from the vessel lumen into the collagen matrix, crossing the endothelial barrier (Fig. 4b). Monitoring the dynamics of cancer cells interacting with the barrier allowed us to distinguish three main types of behavior with (Fig. 4c, top) or without an endothelial barrier (Fig. 4c, bottom). Type I, cancer cells transmigrated from the vessel lumen into the collagen matrix; Type II, cancer cells remained at the endothelial barrier without migrating into the gel; Type III, cancer cells remained in the vessel lumen or, after staying at the endothelial barrier for a certain amount of time, migrated back to the centre of the channel (summarized in Fig. 4d). All three behaviors could occur within the same sample. In the reference system, without HUVECs, cancer cells only demonstrated behavior I and II, where in the type II behavior cells stay at the interface between the channel and the gel. Cancer cells would not migrate back inside the channel once they reached the collagen gel interface. The presence of behavior III only in the microvascular system (which accounts for over 80% trajectory), implies that the endothelial cells forming the endothelial wall acted as a regulated barrier to prevent uncontrolled cancer cell transendothelial migration. Failing to find an accessible region for crossing the barrier, the cells would migrate back towards the vessel lumen. The number of transendothelial migration events from the vessel lumen into the underlying tissue or ECM is a parameter also measured in previous studies^25–30^. The average number of TEM events over time could be related to the average of the total number of cancer cells present in the field of view over time. Figure 5a presents the total numbers of cancer cells that were found to remain overall constant during the duration of the time-lapse experiments in all the samples. Due to cells migrating in and out the FOV, along the channel, a variation of approximately 5 cells was observed for some samples. Therefore, dependence of the TEM events on the total number of cancer cells present in the lumen could be assessed.

From these analyses, the average number ($\frac{{\sum }_{t=1}^{T}\,{n}_{t}}{T}$, where *n*_*t*_ is the number of cells at a particular time frame and *T* is the total number of frames) of cells which transmigrated *versus* the total number of injected cells per FOV, for each sample over 15 hr could be determined (Fig. 5b). The plot in Fig. 5b suggests two types of behavior: first, the number of transmigration does not depend on the amount of cells in the vessel; second, an increasing number of cancer cells could enhance the number of cancer cells that would transmigrate. The ratio of cells that transmigrated over the total number of cells $(\frac{{N}_{av \mbox{-} ext}}{{N}_{av \mbox{-} tot}})$ was calculated to be (0.07 ± 0.02), meaning that about 7% of the total number of cancer cells present in the FOV crossed the endothelium barrier over a 15 hr period of time. Moreover, distance from the barrier was also a key parameter, with cancer cells located further away from the endothelial barrier possessing a lower chance to transmigrate, compared to those in proximity. Thus the number of cells which migrated through was also related to the total number of cells present at the endothelial barrier only. Taking this effect into account, Fig. 5c shows that the total number of cancer cells at the endothelial barrier was found to remain overall constant during the duration of the experiments across all samples. Therefore, the average number of TEM events over time could be related to the average number of cells at the barrier over time (Fig. 5d). By straight-line fitting, we calculated $(\frac{{N}_{av \mbox{-} ext}}{{N}_{av \mbox{-} barrier}})$ to be (0.34 ± 0.08), meaning that about 34% of the number of cancer cells present at the barrier transmigrated over 15 hr.

**Figure 6:**
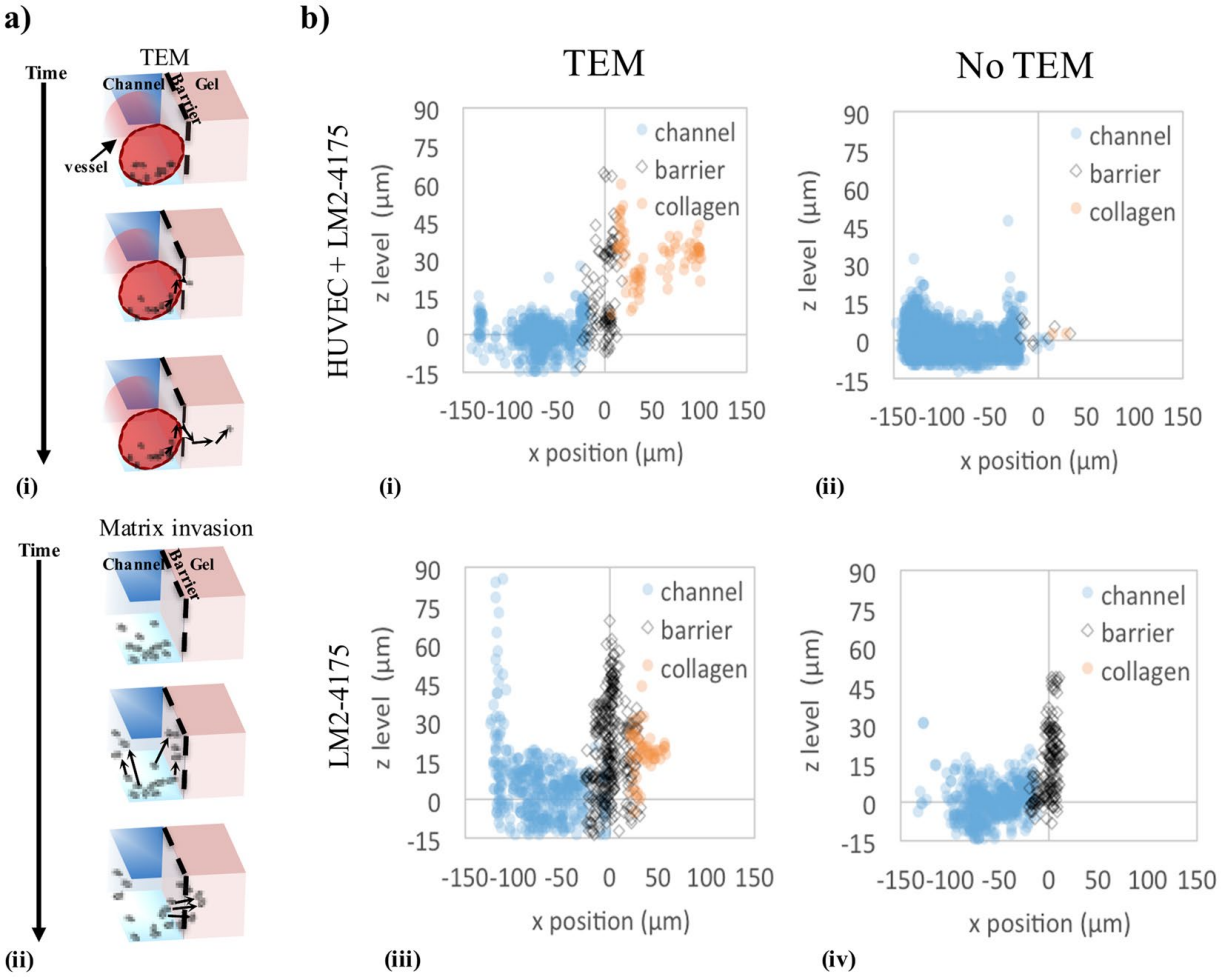
Different mechanisms of trans-interface migration are observed in the endothelial and reference systems. (**a**) Schematic diagrams of cancer transmigration strategies in microvascular (i) and reference (ii) systems. Arrows indicate changes in the cell position over time when transmigration occurs. (**b**) Scatter plots showing the coordinates x (perpendicular to the barrier towards the collagen gel), and z (from the bottom to the top of the channel) of the cancer cells in the three regions. Each dot represents the position of a specific cell at a specific time point. x ≈ 0 corresponds to the position of the endothelial barrier; z ≈ 0 corresponds to the position of the channel bottom wall. (i,iii) Typical samples for the microvascular and reference system, respectively, where transmigration occurred. For the microvascular system, the black dots indicate the occurrence of ‘hot spots’ for cancer cell TEM. (ii,iv) Typical samples for the microvascular and reference system, respectively, where cancer matrix invasion did not occur.

Furthermore, the in-depth image analysis introduced in this platform provides the possibility of tracking the spatial position of the cancer cells in the 3D environment over time. Figure 6 shows a map of the cell population position in the x-z plane (*i*.*e*. the occurrence of the cell positions in the x-z plane over the entire ~15 hr duration of the live-cell imaging), where each scatter point represents a cell’s x-z position at a particular time point. For the microvascular system (i and iii) and the reference system (ii and iv), typical cases with the presence or absence of TEM event are shown. To assist data analysis, we further categorized these data based on whether the cells have eventually entered the channel, barrier or collagen. The data suggests that in the microvascular system (i and ii), the cancer cells mainly stay at the channel bottom due to the effect of gravity (as shown by the high number of scatter points at z = 0, −150 µm ≤ x ≤ 0, where x = 0 is defined as the barrier position). Although in the reference system (iii and iv), the majority of the cancer cells are also found at the channel bottom, they tend to migrate further upwards the collagen surface facing the channel, occupying the entire available interface (as shown by the continuous density of scatter points at x = 0 along z). Trans-interface migration in the reference system seems to occur in a population-driven manner; there is no preferred position for cancer invasion along the collagen interface. In contrast, the microvessel system highlighted distinct ‘hot spots’ for cancer cells to cross the endothelial barrier. There are specific, localized positions for cancer cell attachment at the interface, followed by migration into the collagen from these positions (as shown by the high densities of scatting points for the x ≥ 0 space). Despite much higher probability of cell presence, the cancer cells do not easily transmigrate at the bottom corner of the barrier (which would be the case if the vessel was homogenous in the 3D space). This indicates that cell migration towards these ‘hot spots’ might be directed by certain biochemical signals provided by endothelial cells, which can be sensed some distance away from the original cell positions capable of overriding the strength of biophysical factors (*i*.*e*. gravity). In the work presented here, it is not our intention to elucidate the detailed biochemical mechanism, but rather to propose that variability of microvessel properties across different experimental runs could be a key factor leading to the observed behaviour (see Discussion).

### Cancer cell migration velocity during transendothelial migration and collagen invasion

**Figure 7:**
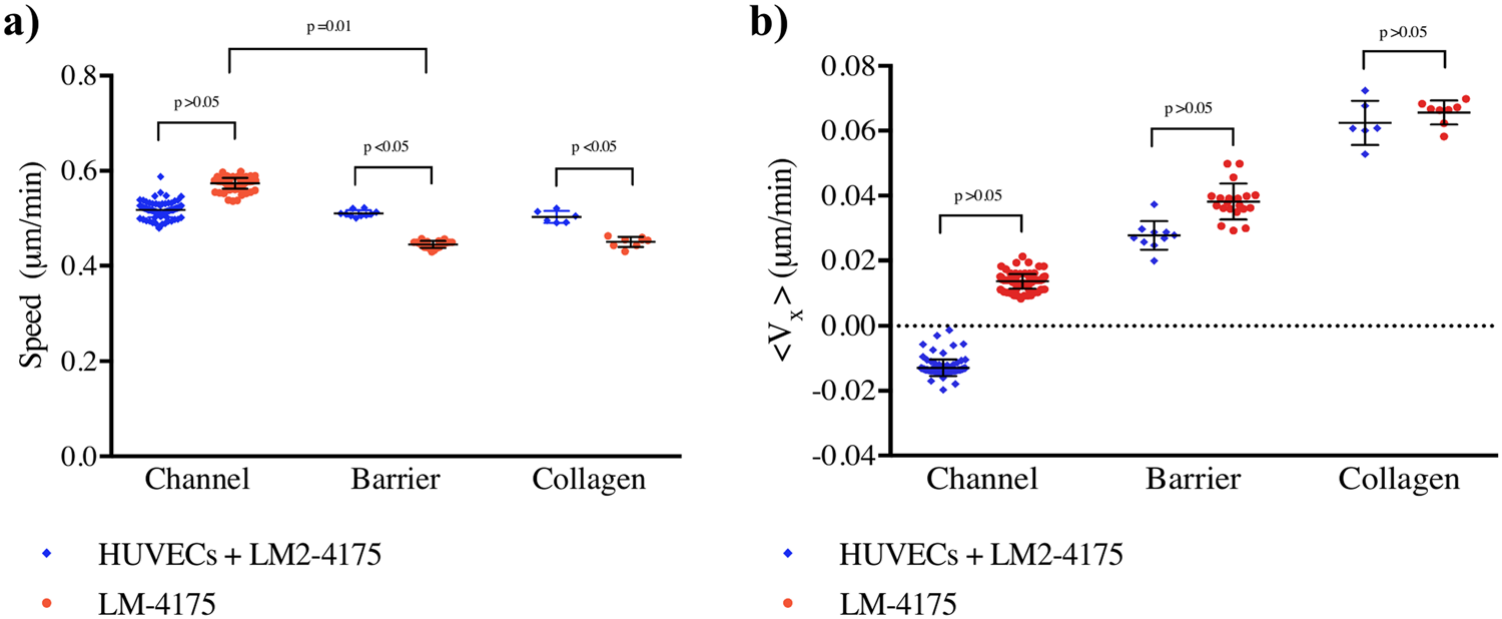
Quantification of cancer cell velocities depicts the effect of the local microenvironment on cancer cell behavior. A total of 6 and 5 samples were analyzed for the microvessel and the reference system, respectively. In each sample, 10–50 cells were tracked (z-stack was acquired) every 20 min over 15 hr. (**a**) Speeds of the cancer cells in the channel, barrier and collagen matrix. (**b**) Cancer cell velocity component along the x-axis (along the channel) was calculated to investigate a preferred direction of migration of cells. All graphs show mean for each group of data points; error bars: SD.

To further explore the possibilities of image-assisted microfluidic platform to study the dynamics of cell movements, the effect of the local microenvironment on cancer cell behavior was investigated by quantifying cancer cell velocity (measured between two consecutive time points, *i*.*e*. 20 min intervals under the imaging conditions) during transmigration and then invasion in three-dimensional collagen. The direction of migration of the cancer cell population inside the microfluidic platform was explored by quantifying the averages of cell velocity components in the x-direction (perpendicular to the barrier, towards the collagen matrix) and y-direction (along the channel), in the microvascular system and in the reference system, in each sample. The speed and velocity components of the cellular population were estimated by calculating a weighted average of all the samples, using the following equations^31^:

$$mean=\frac{{\sum }_{i}\,{w}_{i}\overline{{v}_{i}}}{{\sum }_{i}\,{w}_{i}},\,error=\frac{1}{\sqrt{{\sum }_{i}\,{w}_{i}}},\,weight={w}_{i}=\frac{{n}_{i}}{{\sigma }_{i}^{2}},$$

where $\overline{{v}_{i}}$, *n*_*i*_ and _*σi*_ were the average velocity, number of cells and standard deviation of each sample, respectively (Fig. 7a).

The average cellular speed calculated in the channel, at the barrier and inside the collagen gel remains similar in all regions of the microvascular system (0.52 ± 0.02 μm/min, 0.51 ± 0.01 μm/min and 0.50 ± 0.01 μm/min in the channel, barrier and collagen matrix, respectively, *p* > 0.05); whereas the cell speed decreases when cells migrate from the channel to the barrier in the reference system (0.57 ± 0.01 μm/min, 0.45 ± 0.01 μm/min and 0.45 ± 0.02 μm/min in the channel, barrier and collagen matrix, *p* = 0.01, *p* < 0.05), likely due to the presence of the collagen interface.

Previous studies reported that cancer cells migrate at a velocity of (0.1–1) μm/min, when a mesenchymal migration mechanism occurrs^32,33^. Our results are therefore comparable with other data, and coupled with visual investigation, suggest mesenchymal mode of invasion. The velocity components perpendicular to the barrier, 〈*v*_*x*_〉, was calculated to investigate a preferred direction of migration for the cell population (Fig. 7b). The mean velocity component, 〈*v*_*x*_〉, for the cell population in the three defined regions generated a positive value at the barrier and in the collagen matrix in both the reference and microvascular systems. These quantitative results suggested that cancer cells located at the barrier have tendency to cross the endothelial barrier towards the collagen matrix. Moreover, once inside the collagen gel, the cancer cells tend to migrate further away, towards the centre of the device. Cross-comparing the microvascular and the reference system, similar 〈*v*_*x*_〉 values were obtained in the same microenvironments, for (*p* > 0.05). Although the data presented above might require theoretical analysis and/or it may be necessary to design more experiments to address various questions, our novel approach shows the amount of information that can be extracted from every set of experiments. Thus, we believe our image-assisted microvessel platform is advantageous in comparison to alternative systems, where detailed analysis could not be achieved.

## Discussion

### Fit-for-purpose microvessel application

Commonly used 2D techniques have their limitations, while creating a 3D system must balance a number of trade-offs between the complexity, reliability and physiological relevance of the model compared to the real life cases^34–36^. Microfluidics have allowed researchers to develop advanced 3D assays for cell biology research, in highly controlled microenvironments^37^. With the use of microfluidic platforms, it is possible to incorporate spatiotemporally defined chemical gradients, interstitial flow and shear stresses, and to examine complex interactions among multiple cell types^38,39^. As a result, microfluidics is one of the most promising technologies to develop and optimize *in vitro* cancer models, especially for scientists interested in various steps of metastasis as well as cell-cell interactions occurring during immune responses in malignant tumors.

Table SI.1 in the Supporting information, along with the text below, compare a few models of microfluidic-based vessels, which are used mainly for cancer transmigration studies. All of the described systems present certain advantages and the choice between those platforms should be made based on specific scientific problems that need to be addressed, as well as on how quickly and accurately the experiments could be performed (*i*.*e*. whether the method provides an opportunity for high content studies). Methods of device preparation vary between the groups. An interesting technique for fabricating circular channels which could be adapted for creating microvessels has been described by Abdelgawad *et al*.^40^. However, as previously mentioned, PDMS moulding combined with direct hydrogel injection is currently the most adapted method. The direct hydrogel injection technique involves the injection of ECM-mimicking collagen gel into microfluidic channels that face cell culture channels^29,41,42^. Inside these ‘ECM’ channels, blood vessels, in contact with the collagen gel, can be formed in 1 day; however, these vessels possess a rectangular cross section and their width dimensions are not in the microvessel range. The hydrogel moulding technique is also widely used and was shown by creating microchannels inside hydrogels by using removable rods^15^, sacrificial materials^43,44^ or viscous finger patterning^45^; the latter technique does not allow the formation of vessels that would be ~100 µm in diameter, but the method of creating a microfluidic system is relatively simple. Inside these microchannels, vessels can be formed in 1 day; however, the flow of solution or cell injection inside the vessels requires more sophisticated connections and/or an additional casing unit. The microfluidic platform presented here displays several favorable characteristics for broad user adaptation. A chip with integrated ECM and a rounded, ~100 µm in diameter, microvessel is created with minimised use of reagents and cells, and by following simplified procedure of vessel creation. Moreover, the ability to couple live-cell imaging and image analysis to study dynamics of cell-microenvironment interactions in 3D, significantly increase the utility of the system for bioresearch.

### Potential correlation between ‘hot spot’-dependent TEM and the vessel quality

Our image-assisted microvessel platform enables tracking of single cell migration dynamics in 3D. This leads to mechanistic insights in cell behavior during cancer transendothelial migration, where we observe the presence of ‘hot spots’ in the microvessels. To support our findings, we further provide data from a reference system, where cancer cells were interacting with an endothelial-free channel (Fig. 6). The stark difference in the cellular dynamics between the reference and the microvessel system supports our hypothesis that in presence of endothelial barrier, cells are more likely to search for a region of compromised vessel function, or ‘hot spots’, in order to transmigrate. These kind of experiments cannot be performed in any *in vivo* system, thus they are of interest for future applications which will allow exploration of the interplay between selected microenvironmental factors.

Our work shows that the vessel quality can vary in various experimental runs. Realizing the existence of such vessel variability led us to consider its role for subsequent data interpretation. For example, the number of cancer cells crossing the endothelial barrier over a fixed time period is a commonly measured parameter for TEM studies. Based on this parameter, we suggest that the seemingly two populations of behaviors (i.e. no transmigration *versus* transmigration correlated with the number of cancer cell input) shown in Figs 5d and SI.2, is a result of the vessel quality variability. The presence of transmigration ‘hot spots’ when TEM occurs further supports the above proposition.

To evaluate the effect of vessel barrier function on the cancer cell transmigration, we also used EA.hy926 cell line to act as a comparison to the HUVEC system. EA.hy926 cells are known to have many similar characteristics to HUVECs^46^. The cell line was able to form a confluent 3D microvessel in our microfluidic platform, though it showed the absence of V-cadherin. 70 kDa diffusion measurement yields a value for the EA.hy926 vessel permeability to be 59 ± 12 × 10^−6^ cm/s (n = 4), what is about 5 times higher than permeability of the HUVEC vessel. However, comparing to previous *in vitro* and *in vivo* TEM studies, both the EA.hy926 and the HUVEC microvessel systems reproduce a similar normalized, ensemble average number of transmigration events for comparable breast cancer cell lines (Fig. SI.3). This is despite of the considerable variation in the reported vessel permeability values across different vessel systems (e.g. Fig. SI.1). In summary, any technical or biological constraints associated with our microvessel formation method have resulted in similar transmigration event count as previously reported in literature. It is the ability to track single-cell dynamics which elucidates the existence of TEM ‘hot spots’, and the distinct bi-phasic behaviors shown in Fig. 6. Hence a novelty of our approach is to incorporate very thorough image analysis of the single cell dynamic studies in the microvessel, revealing many details which would otherwise be omitted by analysing an ensemble average.

## Conclusions

This study describes the design of an *in vitro* 3D microvessel which incorporates an endothelial ECM interface. This microvessel-on-a-chip platform can be used to perform dynamic studies of cancer cell behavior during transendothelial migration. The favorable attributes of our microvessel-chip system are: (1) uniform blood vessels of small sizes (~100 µm in diameter), with a rounded cross section, can be fabricated for cancer transmigration studies by following an easy hydrogel injection and cell seeding procedure, with minimised use of reagents and cell, (requiring only ~3 × 10^5^ cells for endothelial tube formation, 2,000–5,000 cells for cancer extravasation, and <10 µL cell solution for each medium exchange); (2) the standardized geometry of the device allows convenient image analysis; (3) dynamics of single-cell behavior can be analysed.

Assisted by image analysis, the statistical distribution of the cancer cell dynamics in the 3D environment was studied over time. Two types of behavior were observed for LM2-4175 seeded inside the microvessel lumen: (1) no noticeable cancer cell transmigration occurred over 15 hr; (2) the number of cancer cells spontaneously crossing the endothelial barrier was positively correlated with the number of cancer cells in proximity to the endothelial/ECM interface. The analysis of the second type of behavior revealed that about 34% of the LM2-4175 cells present immediately adjacent to the barrier (or 7% of the total number of cancer cells present in the field of view) were found to transmigrate within 15 hr.

We believe that the quality of the microvessel lumen could impact which of the two behaviors described above prevails during an experiment. Due to limited assessment capability of the 3D vessel integrity before each experiment, our vessel formation method results in the assembly of microvessels with intact yet varied barrier quality. Despite the differences in microvessel integrity, for all of the experiments performed (including those with lower level of F-actin staining), the presence of endothelial lining in the channel significantly reduced the occurrence of cancer cells at the vessel-collagen I interface region. We are bringing to attention that the integrity of engineered blood vessels created within microfluidic devices may vary, and can be a contributor in observing the diverse transendothelial migration behavior. The findings resulted from the current studies warrant more detailed biological investigation of transendothelial migration events in the future, allowing assessment of many physical features of this complicated process. It may also provide a tractable *in vitro* model for other areas of microvascular research such as immune system-microenvironment interactions^47–49^.

## Electronic supplementary material

Supplementary Information
