# Supplementary material for: Image-Assisted Microvessel-on-a-Chip Platform for Studying Cancer Cell Transendothelial Migration Dynamics
Source: Sci Rep. 2018 Aug 20;8:12480. doi: 10.1038/s41598-018-30776-0 (PMC6102203; doi:10.1038/s41598-018-30776-0)
Supplement: Supplementary file 1 — Supplementary Information [file 41598_2018_30776_MOESM1_ESM.pdf]

Image-Assisted Microvessel-on-a-Chip Platform for Studying Cancer Cell Transendothelial Migration Dynamics

Cristina Bertulli[aΔ], Magda Gerigk[bΔ], Nicholas Piano[b], Ye Liu[b], Duo Zhang[b], Thomas Müller[c,d], Tuomas J Knowles[c,d] and Yan Yan Shery Huang[b*]

[a.] *Cavendish Laboratory, University of Cambridge, Cambridge CB3 0HE, UK*
[b.] *Department of Engineering, University of Cambridge, Cambridge CB2 1PZ, UK*
[c.] *Department of Chemistry, University of Cambridge, Cambridge CB2 1EW, UK*
[d.] *Present address: Fluidic Analytics Ltd., Cambridge CB4 3NP, UK*

[Δ] These authors contributed equally to this project**.**
* corresponding: yysh2@cam.ac.uk

Supplementary Information

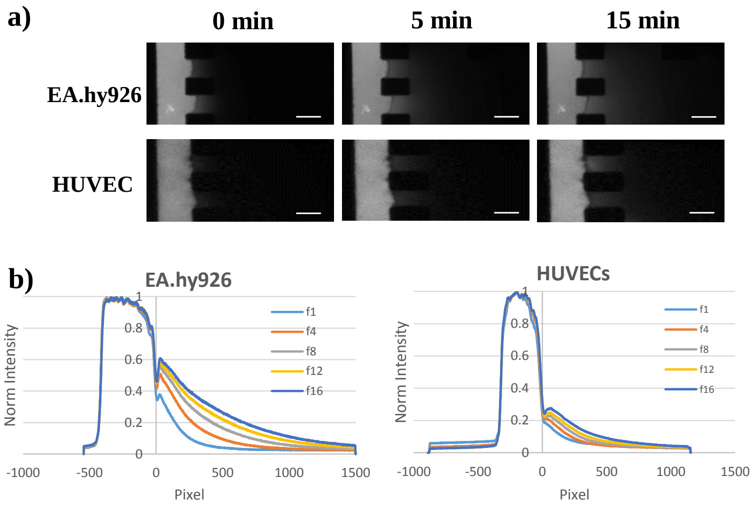

c)

	Zheng et al.	*Zervantonakis et al.*	*Jeon et al.*	*Funamoto et al.*	Current work	Current work
**Cell type**	HUVEC	HUVEC (in presence of cancer cells)	hMVEC	HUVEC	EA.hy926	HUVEC
**Cross-section**	circular	width of 500 µm, height of 120 µm	width of 500 µm, height of 120 µm	width of 500 µm, height of 150 µm	Rounded 120-150 µm	Rounded 100-120 µm
**Collagen concentration [mg/ml]**	6-10	2.5	2	2.5	2	2
**Permeability [x10^{-6} cm/s]**	4.1 ± 0.5	7.5 ± 0.93	3.7 ± 0.59	2.9 ± 1.2	59 ± 12	12 ± 2.4

Figure SI.1: Diffusion measurements for differences in vessel quality between microvessel formed by either EA.hy926 or HUVE cells. a) fluorescent microscope images at 0, 5 and 15 minutes after the injection of 70kDa FITC-dextran for EA.hy926 and HUVE cells. b) Typical x-directional profiles of the normalized fluorescence intensity over 15 min of the infusion 70 kDa FITC-dextran for EA.hy926 (n=4) and HUVECs (n=3. c) comparison of 70 kDa FITC-dextran permeability values for different microfluidic systems between literature and the current work. All scale bars: 100 µm.

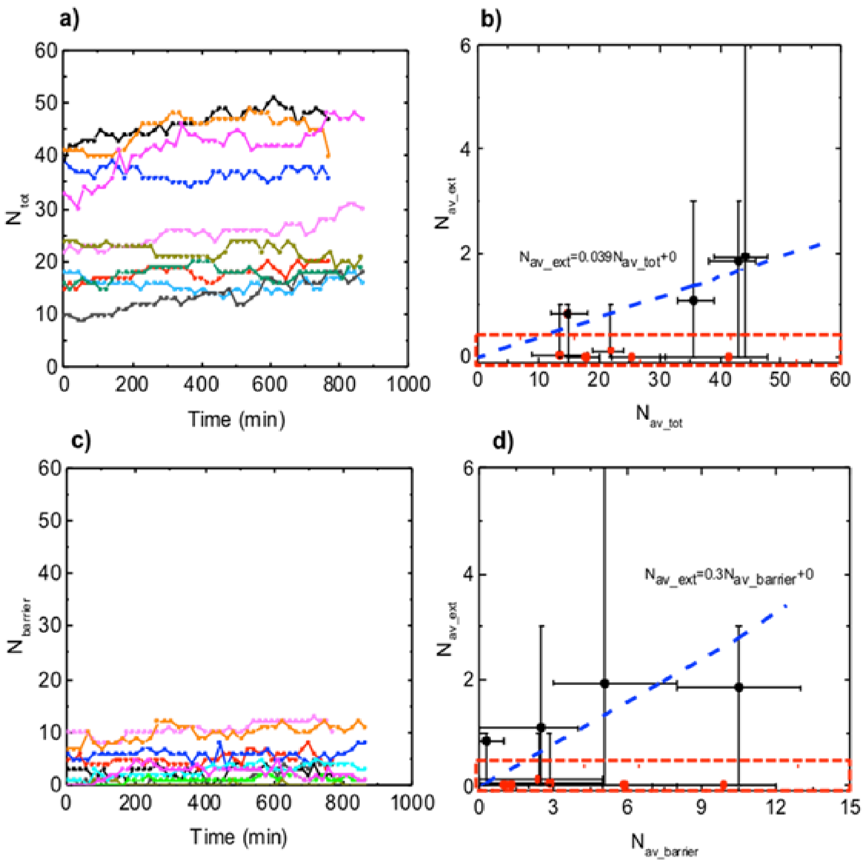

Figure SI.2: Cell number-dependent transendothelial migration in the EA.hy926 microfluidic system. a) Total number of cancer cells (MDA-MB-231) present in the field of view over time, for ten samples. b) The average number of cells which transmigrated through the endothelial barrier *vs* the average number of cancer cells in the channel and at the barrier, for each sample. The error bars indicate the maximum and minimum values of the fluctuation in the cell number over time. The red box indicates that transmigration did not occur. The data points (black dots) were fitted with a straight line with intercept set to zero; the equation of the fitting line is indicated. c) Number of cancer cells present at the barrier over time, for ten samples where cells migrated to the barrier. d) The average number of cells that crossed the endothelial barrier *vs* the average number of cancer cells at the endothelial barrier, for ten samples. The error bars indicate the maximum and minimum values of the fluctuation in the number of cells over time. The red box indicates that transmigration did not occur. The data points indicating TEM events (black dots) were fitted with a straight line with intercept set to zero; the equation of the fitting line is indicated.

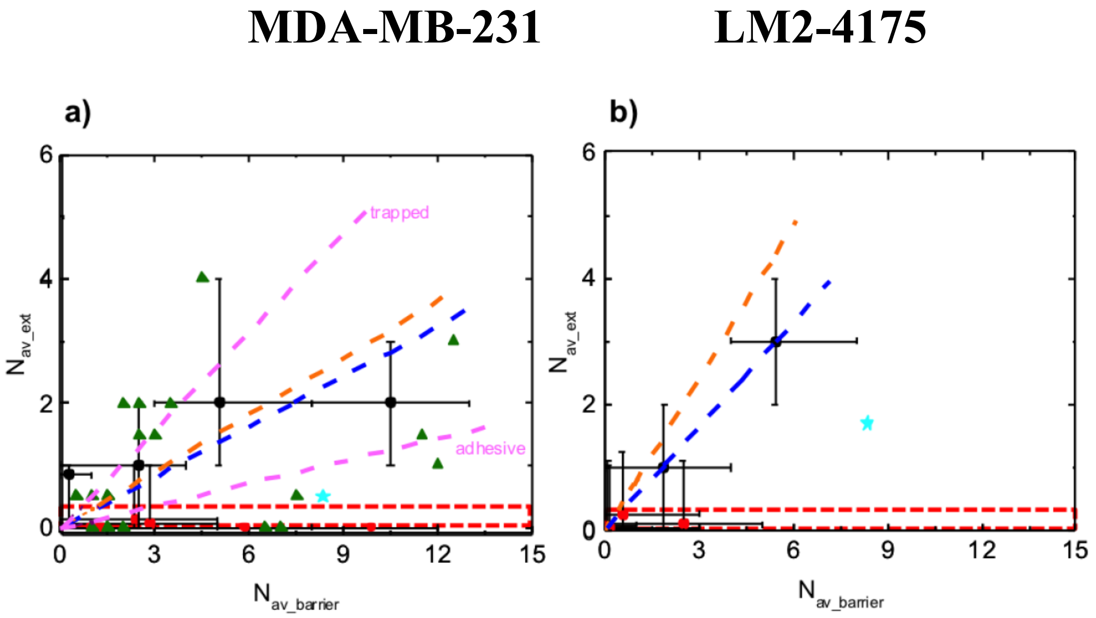

Figure SI.3: Comparison of our data on cancer transendothelial migration, obtained using a microvessel-on-a-chip platform, with *in vivo* and *in vitro* studies performed within 24 hr. a) Plot showing the comparison of our data (red dots and black dots fitted with straight blue line) with *in vitro* microfluidic studies done by Jeon *et al*[29] (triangles) and by Chen *et al*[30] (magenta lines), *in vitro* trans-well studies performed by Gupta *et al*[26] (star) and *in vivo* zebrafish studies performed by Stoletov *et al*[28] (orange line). b) Plot from showing the comparison of our data (red dots and black dots fitted with a straight blue line) with *in vitro* trans-well studies performed by Gupta *et al*[26] (star) and *in vivo* mouse studies done by Gupta *et al*[26] (orange line). The experimental data obtained from other publications were multiplied by the normalisation factor (Area_barrier_study/Area_barrier_paper). Area_barrier is the area of the endothelial barrier interfacing with the collagen matrix; this represents the only accessible area for the cancer cells to transmigrate into the collagen gel. In our study, area_barrier_study=0.015mm^2; in Jeon *et al*[29] area_barrier_paper =0.03 mm^2; in Gupta *et al*[26] area_barrier_paper =0.44 mm^2. For the study of Chen *et al*[30] the slope of the lines (N av_extadhesive=0.1Nav_barrier and N av_exttrapped=0.5Nav_barrier) were calculated considering that 10% of the adhesive cancer cells and 50% of trapped cells that crossed the endothelial barrier, as cited in their work. For the study of Stoletov *et al*[28], the slope of the line (Nav_ext=0.3Nav_barrier) was calculated considering that 30% of the cancer cells extravasated, as cited in their work. For the study of Gupta *et al*[26] the slope of the line (Nav_ext=0.8Nav_barrier) was calculated by considering the different extravasation potential between MDA-MB-231and LM2-4175 cells as cited in their work (0.3*100/35).

	**Yeon *et al.***	**Jeon *et al.***	**Chen *et al.***	**Morgan *et al.***	**Wong & Searson**	**Zheng et al**	**Chrobak et al**	**Current work**
Microvessel-on-a-chip preparation								
**Vessel formation**	self-organization and migration	straightforward seeding	vasculogenesis	straightforward seeding	straightforward seeding	straightforward seeding	straightforward seeding	straightforward seeding
**Cell seeding method**	pipette injection	pipette injection	pressure drop	pipette injection	gravity-driven flow	pipette injection	pipette injection	**pipette injection**
**Type of vasculature**	network	single vessel	network	network	single vessel	network	single vessel	single vessel
**Time of vessel formation (after cell seeding)**	5 days	2 days	2-4 days	24 hr	24 hr	3 days	2 days	16 hr
**Vessel size [µm]**	various (widths of 50 to 200)	width of 120	∅8-96	∅100	∅150	150 x 120	∅100	**∅100**
**Vessel geometry**	random (ladder-type)	standardized	random	standardized	standardized	standardized	standardized	**standardized**
**Co-culture**	possible	possible	possible	possible	possible	possible	possible	possible
Microfluidic platform qualities and properties								
**Type of ECM used**	fibrinogen + aprotinin + collagen type I	collagen type I (2 mg/ml)	fibrinogen (5 or 10 mg/ml)	collagen type I (various concentrations)	collagen type I (7 mg/ml)	collagen type I (6-10 mg/ml)	collagen type I (3 or 6.5 mg/ml)	**collagen type I (2 mg/ml)**
**Life-cell imaging of cellular behaviour**	not incorporated	not incorporated	incorporated (approx. 4 hr)	incorporated (1 hr or 2-3 days)	incorporated (>10hr)	Incorporated	not incorporated	**incorporated (15-16 hr)**
**Cell dynamics analysis**	not incorporated	incorporated	not incorporated	not incorporated	not incorporated	not incorporated	not incorporated	**incorporated**

Table SI.1: Comparison of microfluidic systems created for microvessel formation. All of the presented systems use PDMS-based devices with incorporated ECM.
